# Identification of Novel Breast Cancer Subtype-Specific Biomarkers by Integrating Genomics Analysis of DNA Copy Number Aberrations and miRNA-mRNA Dual Expression Profiling

**DOI:** 10.1155/2015/746970

**Published:** 2015-04-15

**Authors:** Dongguo Li, Hong Xia, Zhen-ya Li, Lin Hua, Lin Li

**Affiliations:** ^1^Institute of Biomedical Engineering, Capital Medical University, Beijing 100069, China; ^2^Institute of Basic Medical Science, Peking Union Medical College, Qinghua University, No. 5 Dong Dan San Tiao, Beijing 100005, China

## Abstract

Breast cancer is a heterogeneous disease with well-defined molecular subtypes. Currently, comparative genomic hybridization arrays (aCGH) techniques have been developed rapidly, and recent evidences in studies of breast cancer suggest that tumors within gene expression subtypes share similar DNA copy number aberrations (CNA) which can be used to further subdivide subtypes. Moreover, subtype-specific miRNA expression profiles are also proposed as novel signatures for breast cancer classification. The identification of mRNA or miRNA expression-based breast cancer subtypes is considered an instructive means of prognosis. Here, we conducted an integrated analysis based on copy number aberrations data and miRNA-mRNA dual expression profiling data to identify breast cancer subtype-specific biomarkers. Interestingly, we found a group of genes residing in subtype-specific CNA regions that also display the corresponding changes in mRNAs levels and their target miRNAs' expression. Among them, the predicted direct correlation of BRCA1-miR-143-miR-145 pairs was selected for experimental validation. The study results indicated that BRCA1 positively regulates miR-143-miR-145 expression and miR-143-miR-145 can serve as promising novel biomarkers for breast cancer subtyping. In our integrated genomics analysis and experimental validation, a new frame to predict candidate biomarkers of breast cancer subtype is provided and offers assistance in order to understand the potential disease etiology of the breast cancer subtypes.

## 1. Introduction

Luminal-A and basal-like subtypes are two major breast cancer subtypes and have shown significant differences in terms of incidence, risk factors, baseline prognosis, age at diagnosis, and response to treatment [1]. Luminal-A breast cancers that express estrogen receptors (ERs) and/or progesterone receptors (PRs) and are negative for human epidermal growth factor receptor 2 (HER2) expression respond well to endocrine therapy and have a generally favorable prognosis [2]. In contrast, the basal-like subtype is of particular clinical focus due to its high frequency, lack of effective targeted therapies, poor baseline prognosis, and tendency to affect younger women [3]. Therefore, the identification of breast cancer subtype-specific biomarkers will help provide biological value for the breast cancer clinical trials and therapy strategies.

Recently, more and more evidence showed that an increasing number of genomic aberrations were observed in the progression from normal sample to tumour sample [4]. Array comparative genome hybridization (aCGH) studies of tumor copy number states have demonstrated that tumors with similar gene expression subtypes may also share similar DNA copy number aberrations (CNA) [5, 6] which can be used to further subdivide expression classes. In the practice, some early aCGH studies on breast cancer found that the highly amplified genes were overexpressed and the highly overexpressed genes were amplified. For example, Pollack et al. found that 62% of highly amplified genes show moderately or highly elevated expression, and DNA copy number influences gene expression across a wide range of DNA copy number alterations [7]. By analyzing the linear and nonlinear relationship between gene copy number and expression, Solvang et al. reveal distinct molecular pathways in breast cancer [8]. Recently, CNA coupled with gene expression has been explored for cancer drivers. For example, Akavia et al. developed a computational framework that integrates chromosomal copy number and gene expression data for identifying known drivers of melanoma and predicted multiple novel tumor dependencies [9]. In addition, cancer subtype-specific biomarkers identification has become the important topic which have also demonstrated diverse prognostic power. For example, Liu et al.'s study revealed that gene expression profiles and clinical features show different prognostic power for the five breast cancer subtypes, and gene expression data of the normal-like subgroup contains more valuable prognostic information and survival associated contexts than the other subtypes [10]. Román-Pérez et al. documented the presence of two distinct subtypes of microenvironment, with active versus inactive cancer-adjacent extratumoral microenvironment influencing the aggressiveness and outcome of ER positive human breast cancers [11]. Therefore, identifying the genes that contribute to the instability of cancer phenotype or cancer subtype by integrating DNA copy number aberrations and gene expression would be useful as a clinical predictor of therapeutic response.

Currently, people have studied the microRNA-gene comodule in cancer patients by integrating multiple genomic data including dual microRNA-gene expression and predicted miRNA-gene interactions. For example, Zhang et al. proposed an effective data integration framework using a multiple nonnegative matrix factorization framework and simultaneously integrated additional network in a regularized manner to identify microRNA-gene regulatory modules associated with ovarian cancer [12, 13]. In particular, it is worth noting that jointly analyzing multiple data types will help enhance the understanding of the role of biomarkers in breast cancer pathogenesis and progression [14]. In our previous studies, we prioritized some breast cancer subtype related biomarkers by analyzing miRNA-mRNA dual expression profiling data [15, 16]. At this time, in our extended study, we conducted an integrative data analysis by combining copy number aberrations and miRNA-mRNA dual expression profiling data to further identify breast cancer subtype-specific biomarkers. Our methods were aimed at the discovery of the interconnected regulatory miRNAs-mRNA pairs as well as the identification of important subtype-specific (luminal-A and basal-like) mRNAs or miRNAs. The simultaneous use of miRNA expression microarray, gene expression microarray, array-CGH, and miRNA-mRNA target relationships can give a more comprehensive understanding of the whole genome of the cancerous cells. Our results have shown that the proposed approach is able to produce meaningful gene regulatory networks that are highly relevant to the biological conditions of the data sets. We found that gene sets residing in subtype-specific CNA regions also display the corresponding changes in mRNAs levels and their counterpart miRNAs expressions. BRCA1-miR-143-miR-145 pairs were selected for further experimental validation and showed positive correlation in breast cancer subtypes.

## 2. Materials and Methods

### 2.1. Data Source

We introduced three data sets into our integrated data analysis. The first dataset included 180 breast cancer subtype samples along with their copy number data. Due to our focus on two distinct breast cancer subtypes (luminal-A and basal-like) in the present study, we selected all of 52 luminal-A samples and all of 40 basal-like samples from this dataset to perform our analysis. All of these samples include Illumina 109,000 SNP marker DNA copy number data. The data are available from the Gene Expression Omnibus series GSE10893 [17]. The second dataset included the mRNA expression levels of 23,256 genes, which are also available from GSE10893. We then selected the mRNA levels of 241 samples (158 luminal-A subtypes and 83 basal-like subtypes). The third dataset reported by Enerly et al. [14] is a miRNA-mRNA dual expression profiling dataset (GSE19536). For this dataset, we selected the miRNA and mRNA expression data of 15 basal-like samples and 41 luminal-A samples. The original miRNA microarrays covered 799 miRNAs arisen from the Agilent Technologies. miRNA expression status was scored as present or absent for each gene in each sample by default settings. miRNAs in samples that were run in replicates were considered present if scored in one of the two arrays. Those miRNAs that were detected in less than 10% of the samples were excluded. This filtering resulted in 489 miRNAs considered to be expressed in this set of human breast tumors. SAM (significance analysis of microarrays) method [18] was used to identify statistically significant differential expression of miRNAs and mRNAs which distinguished the reciprocal basal-like and luminal-A breast cancer subtypes. According to a filtering criterion of *P* < 0.05 and false discovery rates (FDR) < 0.1, 201 differentially expressed miRNAs and 8,796 differentially expressed mRNAs for the third dataset were identified (see our previous study [16]). For each identified miRNA, we obtained its target genes from MicroCosm Targets database (http://www.ebi.ac.uk/enright-srv/microcosm/htdocs/targets/v5/), in which the candidate miRNA-target relationships were mostly predicted by miRanda algorithm [19].

### 2.2. Assessment of Tumor Genomics DNA Copy Number Changes

For the copy number data in the first dataset, we used a sparse Bayesian learning (SBL) model and backward elimination (BE) procedure to find candidate CNA regions. A SBL model was used to find the most likely candidate breakpoints for the copy number state, and the BE procedure was used to remove sequentially the least significant breakpoints estimated by the SBL model, allowing a flexible adjustment of the false discovery rate (FDR) [20]. Therefore, this method provides great flexibility in adjusting the final breakpoint set, and we can obtain a list of breakpoints with lower FDR (<5%) by adjusting the corresponding parameters. We used gada package of R software (http://www.r-project.org/) to implement this analysis.

### 2.3. Determining Subtype-Specific CNAs

To determine subtype-specific CNAs, the segment output file which had arisen from the SBL model and BE procedure was converted into an indicator matrix, where, for each sample, each gene's copy state was represented as −1 = loss, 0 = no change, and 1 = gain. The counts of state for luminal-A subtype and basal-like subtype were compared to identify subtype-specific CNAs. We performed a Chi-square test on the subtype for each gene. Genes with *P* < 0.05 were selected as the subtype-specific genes.

### 2.4. The Expression Change of Genes Residing in Subtype-Specific CNA Regions

Generally, the copy number alteration at gene promoters typically does not alter the coding sequences of genes but contributes to cancer by influencing gene expression [21]. The genes residing in CNA regions are expected to cause the corresponding expression changes. Therefore, genes amplified or deleted as well as overexpressed or underexpressed in a subtype-specific manner are good candidate genes. To determine whether the mRNA levels of the candidate genes residing in subtype-specific CNA regions are correlated with DNA gain or loss, we observed the expression change of these genes between the two subtypes of samples (luminal-A and basal-like). According to the copy state and the expression change of the genes, we divided them into four gene groups: the luminal-A gain group (the significantly high counts of gain and the significantly overexpressed in the luminal-A sample); the luminal-A loss group (the significantly high counts of loss and the significantly underexpressed in the luminal-A sample); the basal-like gain group (the significantly high counts of gain and the significantly overexpressed in the basal-like sample); the basal-like loss group (the significantly high counts of loss and the significantly underexpressed in the basal-like sample). The further analysis will be performed based on these four groups.

### 2.5. The Reconstruction of miRNA-mRNA Dysregulated Relationships

Although some previous studies have identified cancer-related gene subnetworks using the Bayesian approach, these methods did not take advantage of the existing biological knowledge, such as miRNA-mRNA target information [9]. Therefore, in this study, for each of the four gene groups (luminal-A gain, luminal-A loss, basal-like gain, and basal-like loss) obtained from above analysis, we used the Bayesian network to identify breast cancer subtype related biomarkers and their regulatory relationships. In the process of learning a Bayesian network with more nodes, if the search space is not restricted, all of the possible networks will be formed with the variables and this is time-consuming. To address this issue, we reconstructed the miRNA-mRNA target dysregulation relationships as the prior biological knowledge to construct the Bayesian network. In the present study, we used a correlation coefficient ratio (CCR) defined by a previous study [15] to reflect the contrast of miRNA and mRNA target combination strength in two different breast cancer subtypes (luminal-A versus basal-like). The empirical distribution of |CCR| was obtained by using the permutation tests and a threshold value was considered as a cut-off value at a significant level (*P* < 0.05) to screen out the significant miRNA-mRNA dysregulated relationships. If |CCR_*ij*_| is significant and *P*_*ij*-*L*_ < 0.05, we defined the relationship between miRNA *i* and target *j* is luminal-A trend, whereas the relationship is basal-like trend when |CCR_*ij*_| is significant and *P*_*ij*-*B*_ < 0.05, where *P*_*ij*-*L*_ and *P*_*ij*-*B*_ are the *P* values of Pearson correlation coefficients tests for miRNA *i* and target *j* in luminal-A samples and basal-like samples, respectively. The detailed method description is illustrated in our previous study [15].

### 2.6. Construction of Bayesian Networks and Identification of Network Motifs

**Figure 1:**
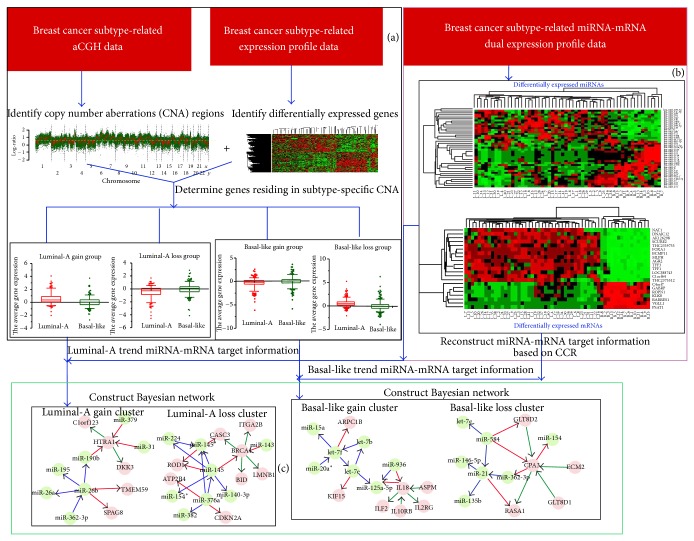
The flow chart of our work is as follows. (a) Using breast cancer subtype related aCGH data and breast cancer subtype related expression profile data to determine the genes residing in subtype-specific CNA regions and divide the genes into four gene groups: luminal-A gain group; luminal-A loss group; basal-like gain group; and basal-like loss group. (b) Using breast cancer subtype related miRNA-mRNA dual expression profile data to reconstruct the miRNA-mRNA target information based on the defined correlation coefficient ratio (CCR) values. (c) Construct Bayesian networks and identify network motifs for the four gene groups: for luminal-A gain and luminal-A loss gene groups, the initial target information was a luminal-A trend. In contrast, the initial target information was a basal-like trend for basal-like gain and basal-like loss gene groups.

For each of the four gene groups (luminal-A gain, luminal-A loss, basal-like gain, and basal-like loss), our goal was to discover the interactions between mRNAs and their regulating miRNAs using their dual expression profiling data under the constraint of the reconstructed breast cancer subtype-trend target information. In order to reduce the search space in the Bayesian network learning process, the reconstructed miRNA-mRNA target information based on CCR was used as the initial structure [22]. For the luminal-A gain and luminal-A loss gene groups, the initial miRNA-mRNA target information was a luminal-A trend. In contrast, the initial miRNA-mRNA target information was a basal-like trend for the basal-like gain and basal-like loss gene groups. A Bayesian network is a graphical model that encodes probabilistic relationships among variables of interest. Let *X* = {*x*_1_, *x*_2_,…, *x*_*n*_} be a set of variables. A Bayesian network over a set of variables is defined as a network structure *S*, which is a directed acyclic graph (DAG) over *X* and a set *P* of local probability distributions. The Bayesian network *S* encodes the assertions of conditional independence; that is, each variable *x*_*i*_ is independent of its nondescendants, given its parents in *S*. In the present study, we allow that the nodes fed into the Bayesian network are genes involved in four gene groups and their regulating miRNAs. The conditional likelihood of the variables given their parents is represented in a Bayesian network by using Gaussian conditional densities. Under the assumption of parameter independence, an initial Bayesian network structure *S* is learned from the training data. From this initial network, greedy search algorithm with random restarts is performed to get the highest score posterior network to avoid local maxima. Finally, an optimized Bayesian network that maximizes the Bayesian factor is obtained using heuristic search of the network space in a specified domain. The Bayesian network learning process was shown in Figure S1 (see Supplementary Material available online at http://dx.doi.org/10.1155/2014/746970). We used BNArray package of R software (http://www.r-project.org/) to construct Bayesian network. Also, we are particularly interested in the network motifs, which are patterns of subgraphs that recur at frequencies much higher than those found in randomized networks [22]. In the present study, network motifs are topological modules which are frequently occurring subgraphs in our integrated regulatory network. Each network motif is composed of multiple interaction types which reflect regulatory, signaling, or compensatory pathway mechanisms using the novel network motif finding algorithm-Cyclus3D [23], and the identified network motifs involve at least two biomarkers. Our work flow chart was shown in Figure 1.

### 2.7. KEGG and CePa Pathway Enrichment Analysis

To further explore each functional gene cluster, we used DAVID (http://david.abcc.ncifcrf.gov/) web tool to perform KEGG pathway enrichment analysis for each of the four gene groups (luminal-A gain, luminal-A loss, basal-like gain, and basal-like loss). A KEGG pathway with a *P* value of 0.01 adjusted by Benjamini [24] correction was considered to be significant. Considering that the network structure information is necessary for the interpretation of the importance of the pathways, we further used a CePa package [25] to perform the extended enrichment analysis by introducing network centralities as the weight of nodes which have been mapped from differentially expressed genes in pathways. Differing from the traditional overrepresentation analysis methods that find significant pathways without the topological information, the CePa takes into account the pathway structure information so that it can capture new findings that are closely related to the current biological problems [25].

### 2.8. The Validation of Predicted Regulatory Relationships of BRCA1-miRNAs Pairs by qRT-PCR Detection

To determine whether BRCA1 regulates miRNA processing, we examined the expression levels of primary, precursor, and mature forms of selected miRNAs using quantitative RT-PCR (qRT-PCR) after overexpression or knock-down of BRCA1 in MCF-7 cells. The major experiment process was described as follows.

*(1) Cell Cultures and Transfection*. MCF-7 breast tumor cell line was provided by the American Type Culture Collection (ATCC). In our study, MCF-7 were cultured in RPMI 1640 media supplemented with 2 mM L-glutamine (Invitrogen), 20 *μ*g/mL gentamycin (Panpharma), 10% fetal bovine serum (Invitrogen), and 0.04 UI/mL insulin (Novo Nordisk) in a humidified atmosphere at 37°C containing 5% CO_2_. MCF-7 cells were transfected with a pCMV6-XL4 vector containing the full-length BRCA1 gene purchased from Origene (Beijing, China) (empty vector as control) or pSilencer siRNA expression vector (Ambion) containing BRCA1 specific shRNA expression cassette (scramble shRNA expression vector as siRNA control). The siRNA target was shown in Table S1. For transfections, cells at 50–60% confluence were incubated with 2 *μ*g of plasmid DNA, using the FuGENE 6 transfection reagent (Roche Molecular Biochemicals, Monza, Italy) according to the manufacturer's instructions. Cells were then selected in G418 (0.4 mg/mL) (Invitrogen Life Technologies, La Jolla, CA, USA). Cell clones that stably expressed G418 and retained growth potential were assayed for BRCA1 expression by qPCR and Western blot assay. qPCR primers for BRCA1 were synthesized as in the following sequence: BRCA1-forward: 5′-TTGTTACAAATCACCCCTCAAGG-3′; BRCA1-reverse: 5′-CCCTGATACTTTTCTGGATGCC-3′. Antibodies for BRCA1 and beta-actin were purchased from Cell Signaling (Danvers, MA, USA).

*(2) RNA Extraction, Reverse Transcription, and qRT-PCR Assays*. Total RNA was isolated from transfected and control MCF-7 cells with TRIZOL reagent (Invitrogen) according to the manufacturer's protocol. The RNA quality was checked by electrophoresis using a Bioanalyzer 2100 with RNA 6000 Nano LabChip and BioSizing A.02.11 software (Agilent Technologies). For mRNA-, pri-, and pre-miRNAs reverse transcription, 5 *μ*g of total RNA was reverse transcribed in a total volume of 15 *μ*L using the First-Strand DNA Synthesis Kit and performed according to the manufacturer's protocol (Amersham Biosciences). For mature microRNA reverse transcription, 5 *μ*g of total RNA was reverse transcribed in a total volume of 15 *μ*L mix with TaqMan microRNA reverse transcription kit (Applied Biosystems). Reverse transcriptase was thermally inactivated (95°C, 10 min). qRT-PCR assays were performed for determining the expression levels of primary, precursor, and mature miRNAs, as described previously [26, 27]. The qPCR reaction was performed using SYBR Green PCR Master Mix (Applied Biosystems) for pri- and pre-miRNAs. For detection of mature miRNAs, TaqMan MicroRNA assay kit (Applied Biosystems) was used, according to the manufacturer's protocol. The TaqMan reaction was performed with TaqMan Fast Universal PCR Master Mix (Applied Biosystems). Data analysis was performed using the comparative Ct method. Results were normalized to human beta-actin for pri- and pre-miRNAs or human U6 small nuclear RNA (snRNA; hRNU6-1) for mature miRNAs.

*(3) In Vivo Monitoring of Pri-miRNA Processing*. Plasmid constructs with pri-miRNA at the 3′ untranslated region of firefly luciferase cDNA and BRCA1 or siBRCA1 expression vectors were transfected into MCF-7 cells. Cell extracts were prepared at the 48-hour point after transfection, and the ratio of firefly and* Renilla* luciferase was measured using a Dual-Luciferase Reporter Assay system (Promega). The values were further normalized by using an empty pmirGLO vector and are indicated with standard deviation. The values are presented as the mean ± SD of results of separate experiments and were compared using Student's *t*-test. Values at *P* < 0.05 were considered to indicate significant differences. All analyses were conducted using JMP IN software, version 5 (SAS).

## 3. Results

### 3.1. Subtype-Specific CNAs Were Determined by Assessment of Tumor Genomics DNA Copy Number Changes

By implementing the SBL model and backward elimination (BE) procedure, we obtained the candidate CNA regions. In regard to the luminal-A subtype, the total counts of gains and losses were 3,650 and 3,544, respectively. For the basal-like subtype, the total counts of gains and losses were 4,024 and 3,980, respectively. In particular, the highest counts of CNA regions for a luminal-A subtype sample and a basal-like subtype sample were 663 and 547, respectively (see Figure S2). Moreover, the copy number changes detected high frequency regions (more than 40% across the breast cancer subtype samples) included 8 regions (2 gain regions and 6 loss regions) for the luminal-A subtype and 18 regions (6 gain regions and 12 loss regions) for the basal-like subtype, respectively (see Table 1). Peak incidences were observed in smaller subregions, that is, gains of 10q11-q27 (62.5%) for basal-like subtype and losses of 17q21 (59.60%) for luminal-A subtype. These regions have all been previously shown to be associated with subtype related breast cancers.

In this analysis, the counts of state for subtype were compared to identify the subtype-specific CNAs. To avoid loss, the multiple test corrections were not performed and 4,551 genes with *P* < 0.05 were then gathered for each subtype. To determine whether the mRNA levels of these candidate genes residing in subtype-specific CNA regions were also correlated with DNA gain or loss, we observed the expression change of these genes between the two distinct samples (luminal-A and basal-like). The results indicated that 1,267 genes had the simultaneous subtype-specific CNAs as well as the corresponding expression change. According to the counts state and the expression change of genes, we divided the genes into four gene groups as the method described: luminal-A gain (261 genes), luminal-A loss (297 genes), basal-like gain (298 genes), and basal-like loss (411 genes) (see Figure S3).

### 3.2. Several Breast Cancer Related Regulatory Network Motifs Were Identified Based on Bayesian Networks and Reconstruction of miRNA-mRNA Dysregulated Relationships

To acquire the prior miRNA-mRNA target information as the initial structure of the Bayesian network, we reconstructed the miRNA-mRNA target dysregulated relationships based on the defined CCR. The CCR critical value is of 6.17, and, by using this criterion, 2,659 luminal-A trend and 3,563 basal-like trend miRNA-target dysregulated relationships were identified (see our previous study [15]).

**Figure 2:**
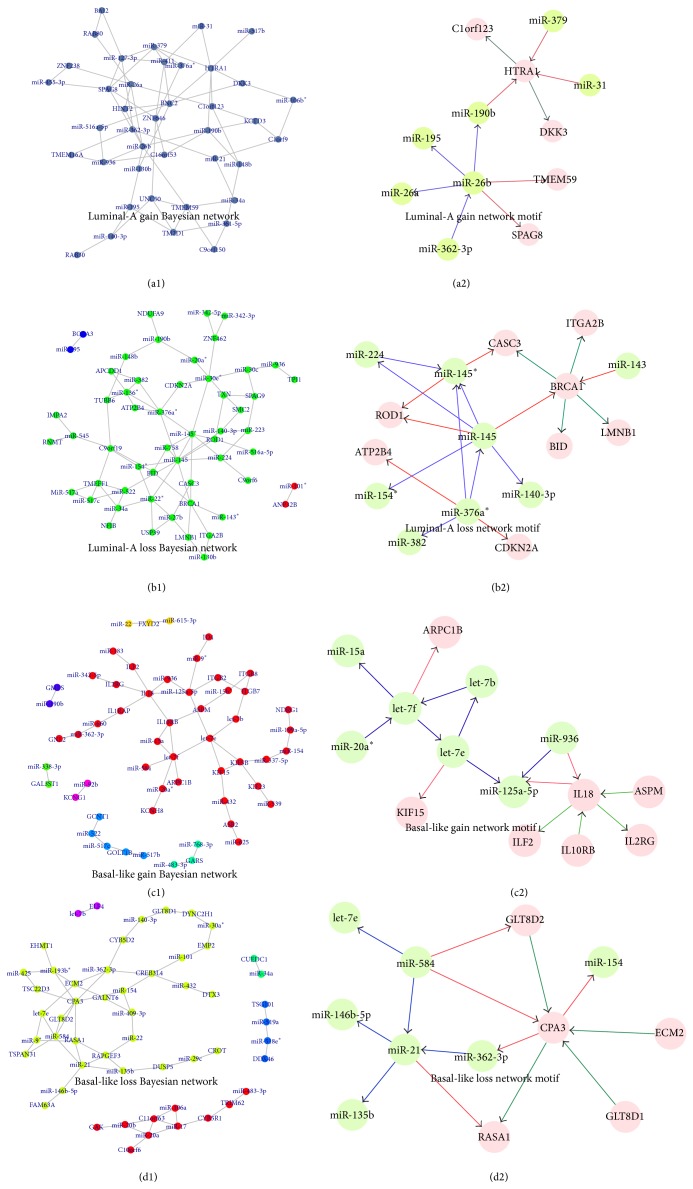
Bayesian network and network motifs. (a1) Luminal-A gain Bayesian network; (a2) luminal-A gain network motif; (b1) luminal-A loss Bayesian network; (b2) luminal-A loss network motif; (c1) basal-like gain Bayesian network; (c2) basal-like gain network motif; (d1) basal-like loss Bayesian network; and (d2) basal-like loss network motif. In (a2–d2), each blue line indicates the interaction between two miRNAs; each red line indicates the relationship between miRNA and its target; and each green line indicates the interaction between two genes.

For each of the four gene groups, the nodes fed into the Bayesian network contain the following: (1) luminal-A gain group: 73 nodes involved in miRNA-mRNA luminal-A trend pairs; (2) luminal-A loss group: 81 nodes involved in miRNA-mRNA luminal-A trend pairs; (3) basal-like gain group: 54 nodes involved in miRNA-mRNA basal-like trend pairs; and (4) basal-like loss group: 67 nodes involved in miRNA-mRNA basal-like trend pairs. We utilized their miRNA-mRNA dual expression profiling data to construct the Bayesian networks (see Figures 2(a1)–2(d1) and Table 2). From Table 2, it is evident that the luminal-A loss gene group has a greater betweenness and clustering coefficient when compared to the other three gene groups. That means the genes (miRNAs) involved in the luminal-A loss group are on higher number of shortest paths between partners and tend to interact with each other. In particular, we observed that miR-145 displayed the highest degree in the network. Blenkiron et al. [28] have previously observed higher expression of miR-145 in luminal-A samples, and miR-145 may be one of the miRNAs related to the breast cancer subtype. In addition, the identified let-7e for the basal-like gain group and miR-21 for the basal-like loss group are all approved to be breast cancer subtype related [29, 30]. Furthermore, we used the Cyclus3D algorithm to identify the network motifs that are patterns of subgraphs that recur at frequencies much higher than those found in randomized networks (see Figures 2(a2)–2(d2)). For each of the network motifs, we calculated the Pearson correlations for each of the links. The results showed that all of the links in these network motifs are significant (*P* < 0.05). The average Pearson correlation coefficients were 0.527 for the luminal-A gain group, 0.495 for the luminal-A loss group, 0.658 for the basal-like gain group, and 0.701 for the basal-like loss group, respectively. Although the results generated by our method are compact with only a small number of interactions, some of the identified miRNAs (or mRNAs) by our integrated data analysis are particularly breast cancer related. As an example, in the luminal-A loss related Bayesian network motif, BRCA1 connected multiple significantly underexpressed miRNAs or genes in basal-like samples. It has been reported that breast cancers in BRCA1 mutation carriers frequently have a distinctive basal-like phenotype. A new finding has supported a derivation of the majority of human BRCA1-associated and sporadic basal-like tumors from luminal progenitors rather than from basal stem cells [31]. For another example, in basal-like loss related Bayesian network motif, given the importance of miR-21 in tumorigenesis, Yang et al. found that miR-21 affects the expression of many of its targets through translational inhibition by knocking down the expression of endogenous miR-21 in MCF-7 breast cancer cells [32].

**Figure 3:**
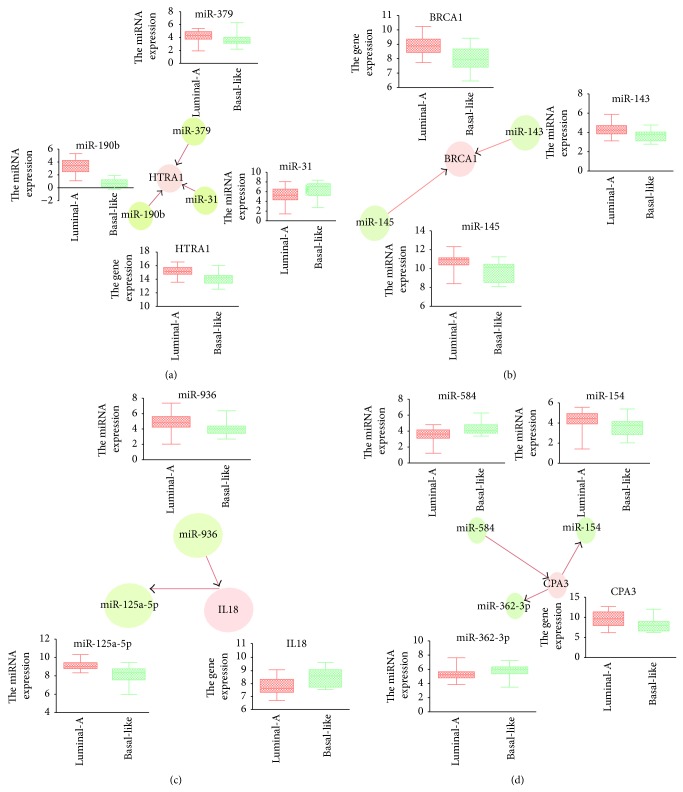
Genes targeted by differentially expressed miRNAs in four network motifs. (a) Luminal-A gain network motif; (b) luminal-A loss network motif; (c) basal-like gain network motif; (d) basal-like loss network motif.

**Figure 4:**
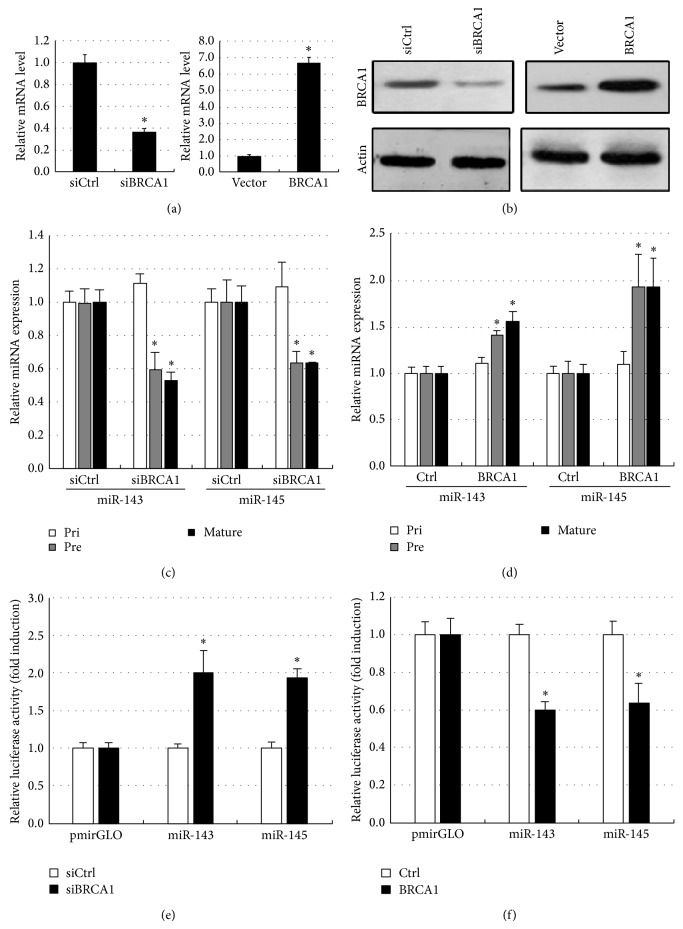
BRCA1 facilitates miR-143 and miR-145 processing. In order to confirm whether BRCA1 facilitates miR-143 and miR-145 processing, BRCA1 overexpression and siBRCA1 expression plasmids were transfected into MCF-7 cells. The empty vector or scramble siRNA expression vector served as controls. (a) BRCA1 mRNA level and (b) protein level were examined by qPCR and Western blot, respectively, in stable transfected cells, normalized by beta-actin (^∗^ *P* < 0.05 as compared with mock control; *n* = 3). Then, the expression levels of the primary (pri), precursor (pre), and mature (mat) forms of the indicated miRNAs were examined in human BRCA1 (c) overexpressed and (d) knock-down MCF-7 cells using qRT-PCR analysis. Pri- and pre-miRNAs were normalized by beta-actin, and mature miRNA was normalized by U6 snRNA (^∗^ *P* < 0.05 as compared with mock control; *n* = 3). Meanwhile, in vivo monitoring assay of pri-miRNA processing in (e) BRCA1 overexpression or (f) knock-down MCF-7 cells carrying miR-143 or miR-145 at the 3′ untranslated region of the luciferase gene. The intensities were normalized by* Renilla* luciferase and are shown as fold induction as compared with an empty pmirGLO vector (^∗^ *P* < 0.05; *n* = 3). Error bars represent standard deviation.

It should be noted that some interactions have been shown to be highly relevant to the subtype of breast cancer, and several of the miRNAs involved in the interactions have been confirmed to be breast cancer subtype related using evidence from previous studies. For example, in the luminal-A gain related Bayesian network motif, we found that HTRA1 was regulated by multiple biomarkers including three miRNAs (miR-379, miR-190b, and miR-31) and two genes (DKK3 and C1orf 123), where miR-190b and miR-379 showed a higher expression in the luminal-A subtype than found in the basal-like subtype, whereas miR-31 presented the contrary situation (see Figure 3(a)). Recent evidences approved that HTRA1 might function as a tumor suppressor by controlling the epithelial-to-mesenchymal transition and might function in chemotherapeutic responsiveness by mediating DNA damage response pathways by characterizing expression in primary breast tissues and the seven human breast epithelial cell lines [33]. In the luminal-A loss related Bayesian network motif, BRCA1 showed the most links with other biomarkers, including two miRNAs (miR-143 and miR-145) and 4 genes (CASC3, ITGA2B, BID, and LMNB1). BRCA1, miR-143, and miR-145 all displayed the higher expression in the luminal-A subtype than in the basal-like subtype (see Figure 3(b)). This suggests that there might be a positive correlation between BRCA1 and miR-143-miR-145. To validate whether BRCA1 regulates these two miRNAs, we examined the expression levels of primary, precursor, and mature forms of miR-143 and miR-145 using quantitative RT-PCR (qRT-PCR) after overexpression or knock-down of a BRCA1 in MCF-7 cells (see Figures 4(a) and 4(b)). Plasmid constructs with pri-miRNA at the 3′ untranslated region of firefly luciferase cDNA (pmirGLO-miR-143, pmirGLO-miR-145) and BRCA1 or siBRCA1 expression vectors were transfected into MCF-7 cells. The primer sequences used for cloning are shown in Table S1. We found that BRCA1 increased precursor and mature miRNAs level of miR-143 and miR-145, though their primary transcripts were found to be with no significant changes (see Figure 4(c)). On the contrary, knock-down of BRCA1 attenuated the expressions of precursor and mature forms of miR-143 and miR-145, whereas their primary transcripts were found to have no significant changes (see Figure 4(d)). Next, to determine whether BRCA1 processes a pri-miRNA substrate, we performed an in vivo pri-miRNA processing monitoring assay of BRCA1 function as previously described [34]. MCF-7 cells were transfected with a luciferase vector construct carrying a segment of pri-miR-143 and pri-miR-145 between the luciferase gene and polyadenylation signal. By using this monitoring system, we observed that BRCA1 overexpression caused a decrease in luciferase activity containing pri-miR-143 and pri-miR-145 sequences (see Figure 4(e)), whereas that activity was increased by knockdown of BRCA1 (see Figure 4(f)). Collectively, these results demonstrate that BRCA1 enhances miR-143 and miR-145 processing of human breast cancer-associated specific miRNAs in vivo.

Besides miR-145, in the basal-like gain related Bayesian network motif, IL18 was found to be regulated by two miRNAs (miR-936 and miR-125a-5p) and four genes (ASPM, ILF2, IL10RB, and IL2RG). We found that two miRNAs displayed the higher expression in the luminal-A subtype than in the basal-like subtype (see Figure 3(c)). Recent studies have shown that the expression of miR-125a-5p is downregulated in human breast cancer and also that a germline mutation in mature miR-125a-5p is closely associated with breast cancer tumorigenesis [35, 36]. In the basal-like loss related Bayesian network motif, CPA3 was regulated by three miRNAs (miR-154, miR-362-3p, and miR-584) and four genes (GLT8D2, GLT8D1, ECM2, and RASA1). Except miR-154 showing the higher expression in the luminal-A subtype than in the basal-like subtype, other two regulating miRNAs (miR-362-3p and miR-584) showed the lower expression in luminal-A subtype samples (see Figure 3(d)). This is supported by the report showing that the expression levels of miR-154 are negatively correlated with Estrogen receptor (ER) positivity in a cohort of early breast cancers [37].

### 3.3. Functional Enrichment Analysis for Four Gene Groups or Network Motifs

**Figure 5:**
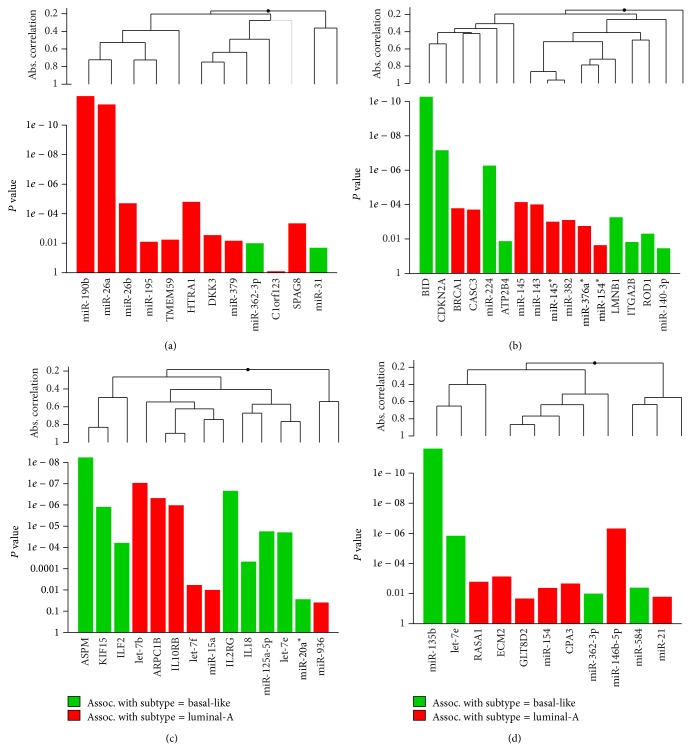
Global test for 4 identified network motifs. (a) Luminal-A gain network motif; (b) luminal-A loss network motif; (c) basal-like gain network motif; (d) basal-like loss network motif. This graph is based on the decomposition of the test statistic into the contributions made by each of the genes (or miRNAs) in the alternative hypothesis. The graph illustrated the *P* values of the tests of individual component genes (or miRNAs) of the alternative. The plotted genes (or miRNAs) are ordered in a hierarchical clustering graph and the clustering method is average linkage.

For each of the gene groups (luminal-A gain, luminal-A loss, basal-like gain, and basal-like loss), we performed KEGG and CePa enrichment analysis. The results were shown in Table 3. From Table 3, the CePa enrichment analysis results showed that only genes of the luminal-A gain group and basal-like gain group were enriched on the significant pathways. It is interesting here to note that the significantly enriched pathway of the luminal-A gain genes group is pancreatic cancer (*P* = 0.0093). Although the pancreatic cancer pathway appears not to be associated with breast cancer, some reports have determined that mutations in the BRCA2 gene have been implicated in pancreatic cancer susceptibility through studies conducted on high-risk breast and ovarian cancer families. A recent study suggested that BRCA2 mutations could account for 6% of moderate and high-risk pancreatic cancer families [38]. In addition, there were two significantly enriched pathways of the basal-like genes group: alanine, aspartate, and glutamate metabolism pathway (*P* = 0.0052) and cytokine-cytokine receptor interaction pathway (*P* = 2.85*E* − 04). This result indicated that the genes involved in the basal-like group were significantly enriched on the functions related to amino acid metabolism and cytokine-cytokine receptor interaction. In fact, many studies have reported that the genes differentially expressed in breast cancer cells are more inclined to be enriched on cytokine-cytokine receptor interaction pathway [39, 40]. Also, to further explore whether the identified four network motifs are associated with the breast cancer subtype, we used Goeman's global test here to determine their significance. The global test potentially can determine whether the global expression pattern of a group of genes and miRNAs is significantly related to the clinical outcome [41]. The results showed that the identified four network motifs are all strongly associated with the breast cancer subtype (*P* = 4.89*E* − 16 for the luminal-A gain network motif; *P* = 1.40*E* − 13 for the luminal-A loss network motif; *P* = 2.14*E* − 14 for the basal-like gain network motif; and *P* = 1.07*E* − 15 for the basal-like loss network motif; see Figure 5). From Figure 5, we can see that some miRNAs displayed a strong association with a particular breast cancer subtype, such as miR-135b which has been proven to be upregulated in basal-like tumor subtypes [14].

### 3.4. The Identified Four Network Motifs by Kaplan-Meier (KM) Survival Analysis

**Figure 6:**
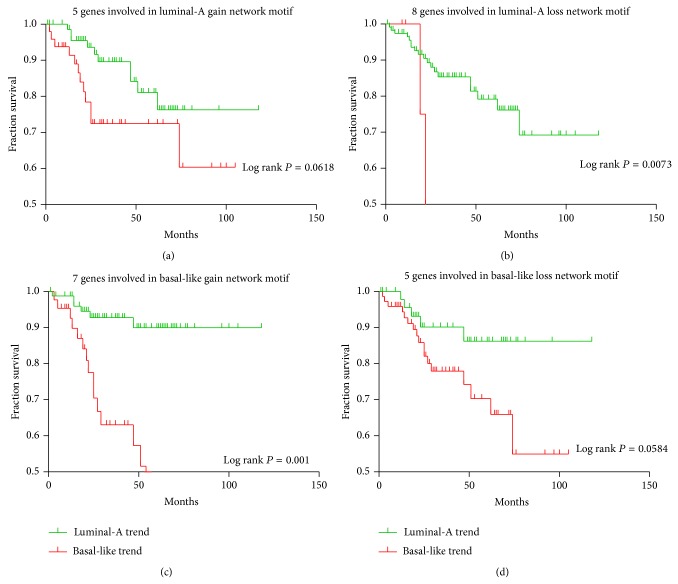
Kaplan-Meier (KM) survival analysis for genes involved in the identified network motifs. (a) A group of genes involved in the luminal-A gain network motif; (b) a group of genes involved in the luminal-A loss network motif; (c) a group of genes involved in the basal-like gain network motif; (d) a group of genes involved in the basal-like loss network motif.

To explore whether the global expression pattern of the identified four network motifs or gene groups involved in these network motifs are significantly correlated with survival, we performed a Kaplan-Meier (KM) survival analysis using the mRNA expression levels of the second dataset and the miRNA-mRNA dual expression levels of the third dataset, respectively. In this analysis, each network motif or gene group was classified using *K*-means clustering (*k* = 2) based on the miRNA or mRNA expression levels into two groups which were defined as either luminal-A trend or basal-like trend according to the proportion of two breast cancer subtype samples. In other words, if the predicted group acquired with *K*-mean cluster includes a greater number of luminal-A samples than basal-like samples, this group is defined as luminal-A trend and vice versa. We performed the Kaplan-Meier (KM) survival analysis for these network motifs (for the third dataset) or gene groups involved in the network motifs (for the second dataset) and assigned KM *P* values for each of the sets in order to stratify the patients into survival groups on the basis of the identified sets. For the second dataset in which only mRNA expression values were included, the analysis found that the genes groups extracted from the luminal-A loss network motif and the basal-like gain network motif displayed the significantly different survival rates (log rank *P* = 0.0073 and log rank *P* = 0.001, resp.). Although we cannot see the rest of the two gene groups extracted from the luminal-A gain network motif (log rank *P* = 0.0618) and the basal-like loss network motif (log rank *P* = 0.0584) show the significant survival curve differences, we believe an increased sample size might change these results. Interestingly, for all of the four gene groups, the basal-like trend samples were associated with the worse outcomes and lower survival rates compared to the luminal-A trend samples (see Figure 6). This is supported by the previous study in which the basal-like breast cancer subtype was approved to have a poor prognosis compared with the luminal-A and luminal-B subtypes [42]. However, for the third dataset in which both miRNA and mRNA expression values were included, we did not find any significant association of any of the identified network motifs: log rank *P* = 0.6798 for the luminal-A gain network motif; log rank *P* = 0.4074 for the luminal-A loss network motif; log rank *P* = 0.7521 for the basal-like gain network motif, and log rank *P* = 0.5020 for the basal-like loss network motif, which might be caused by the small sample size of this dataset.

Moreover, we additionally performed Kaplan-Meier (KM) survival analysis for each of the 25 genes involved in the identified four network motifs. We divided the sample into high expression group when its gene expression value is higher than the median expression. On the contrary, the sample is divided into the low expression group. As a result, 5 genes (SPAG8, KIF15, ILF2, GL8TD1, and ASPM) were found to have a significant log-rank *P* value in patient stratification (high expression group versus low expression group). We also found that the low average expression of SPAG8 and GL8TD1 was associated with the worse outcomes when compared to the high expression (see Figure S4(A) and 4(D)). A contrary trend was observed with KIF15, ILF2, and ASPM (see Figures S4(B), 4(C), and 4(E)). Some of these results can be confirmed by recent studies. For example, a recent study found that ASPM displayed obvious differential expressions in different breast cancer subtypes, and these expression levels were associated with the clinical outcomes [43]. Moreover, our study additionally detected a significant correlation between the expression of the ASPM and CCNB2, and the elevated cytoplasmic CCNB2 protein levels were confirmed to be strongly associated with the short-term disease-specific survival of breast cancer patients [44].

## 4. Discussions

In the past few years, more and more evidence has shown the relationships between biomarkers and cancers. The significant signatures of mRNA or miRNA expression profiles can be linked to various types of tumors, thereby suggesting that mRNA or miRNA profiling has diagnostic and prognostic potential. As one remarkable feature, increasing number of genomic aberrations has been observed in the progression from normal sample to disease sample. DNA copy number was found to influence gene expression across a wide range of DNA copy number alterations, and it has been reported that at least 12% of all variation in gene expression among breast tumors was directly attributed to variation in gene copy number [8]. The significant CNA of a potential gene should also be reflected in the expression of its mRNA. Previous and recent whole-genome analyses of copy numbers and gene expression have led to the identification of global cellular processes which are underlying malignant transformation and progression of breast cancer subtypes. Some studies have confirmed that the basal-like subtype was the most distinct in cases with common losses of the regions containing the greatest overall genomic instability. Therefore, application of aCGH allows a direct coupling to the copy number changes with the potential target genes of miRNAs.

In the present study, we provided an integrative data analysis by combining the copy number aberrations and miRNA-mRNA dual expression profiling data to construct regulatory networks from multiple sources of data: copy number data, gene expression profiles of miRNAs and mRNAs, target information based on sequence data, and sample categories. Our study takes into account not only the association between the copy number change and gene expression, but also the association between the expression of miRNAs and their targets. Specifically, we found some breast cancer subtype related genes and miRNAs through the identified network motifs, and these identified biomarkers might be potential targets for the subtype diagnosis and therapy. In the practice, data integration analysis can identify some important biomarkers which cannot be found by many simplistic approaches. Therefore, the clinical subtype-specific driver networks identified through data integration are reproducible and functionally important. Our integrated data analysis can assist in revealing important findings with regard to the underlying molecular mechanisms of breast cancer subtypes. Some discovered interactions and molecular functions have been confirmed by breast cancer documented related study results. In particular, our experimental validation showed the positive correlation for BRCA1-miR-143-miR-145 pairs in breast cancer subtypes. In addition, many of the other discovered biomarkers are of high statistical significance and thus are strong candidates for validation by future experiments.

However, the limitations of our analysis should also be noted. In our analysis, those genes showed copy number abnormalities but did not display the significant over- or underexpression in different subtypes that were excluded. It is important to note that a recent study observed some subtype-specific genes that had no significant CNA, or a relatively poor correlation between CNA and gene expression [45]. Moreover, we only analyzed the predicted direct miRNA-target regulation, due to the computational complexity. Many other relationships such as the protein-protein interaction information (PPI) and the transcription regulation network are not included. On the other hand, the lack of miRNA-mRNA dual expression profiling datasets causes the limitations in the data and these results must be confirmed in the future studies when more miRNA-mRNA dual expression profiling datasets are available. Additionally, it should be noted that, except for the copy number changes and miRNA-mRNA dual expressions, the mutations, protein-protein networks, methylation alterations, and histone modifications can also influence the integrated data analysis results. Therefore more datasets and more biological knowledge are needed to validate the results, and our future study will combine large-scale data from a variety of analyses at the SNP, gene, methylation alteration, and protein levels. This will assist directly towards better understanding of disease pathology. Furthermore, the amplifications and homozygous deletions are relatively small regions, which may be overlooked by CGH techniques. The latest new technique laser microdissection applied for the vast majority of cases will obtain a much higher percentage of cells allowing a more reliable detection of copy number changes, which will be utilized in our future study.

In summary, some of identified biomarkers have been implicated in breast cancer subtypes and might play important roles in breast cancer subtypes since they are considered therapeutic targets. Therefore, the joint analysis of array comparative genomic hybridization (aCGH) copy number data and microarray gene expression data will dissect biological relationships relevant to our understanding of breast cancer subtypes. This may assist in revealing important findings as well as identifying candidate breast cancer subtype related biomarkers by using the constructed biological networks with regard to the underlying molecular mechanisms of breast cancer subtypes.

## Supplementary Material

Figure S1 provided the dynamic Bayesian network construction process. Figure S2 provided the highest counts of CNA regions for a luminal-A subtype sample and a basal-like subtype sample. Figure S3 is the box plots displaying the average gene expression of four gene groups (luminal-A gain, luminal-A loss, basal-like gain and basal-like loss). Figure S4 provided the Kaplan-Meier survival analysis results for genes involved in the identified four network motifs. Table S1 provided the siRNA target and the primer sequences used for cloning.

## Figures and Tables

**Table 1:** The detected high frequency CNA regions (more than 40% across samples).

Gains			Losses		
Subtype	Chromosome	Frequency (%)	Subtype	Chromosome	Frequency (%)
Luminal-A	5p15.3-q11.1	46.20	Luminal-A	1p36.3-31.2	55.80
Luminal-A	16p13.3-13.1	61.50	Luminal-A	11p15.5-15.4	42.30
Basal-like	2p25.3-24.2	42.50	Luminal-A	17p13.3-11.2	59.60
Basal-like	6p25.3-21.2	52.50	Luminal-A	18p11.3-q12.1	40.40
Basal-like	9p24.3-22.1	47.50	Luminal-A	19p13.3-13.2	42.30
Basal-like	10p15.3-11.1	62.50	Luminal-A	22q11.1-13.1	50.00
Basal-like	12p13.3-13.1	47.50	Basal-like	1p36.3-35.3	55.00
Basal-like	21q11.1-21.3	60.00	Basal-like	3p26.3-25.2	42.50
			Basal-like	4p16.3-15.3	50.00
			Basal-like	7p22.3-21.3	45.00
			Basal-like	8p23.3-23.1	67.50
			Basal-like	11p15.5-15.2	57.50
			Basal-like	13q11-14.3	57.50
			Basal-like	14q11.2-q13.1	42.50
			Basal-like	16p13-11.2	45.00
			Basal-like	17p13.3-11.2	67.50
			Basal-like	19p13.3-13.1	65.00
			Basal-like	22q11.1-12.1	50.00

**Table 2:** The topological properties of Bayesian networks for four gene groups.

Groups	Avg. degree	Avg. betweenness	Avg. clustering coefficient	Avg. closeness	Avg. number of neighbors	Network density	Network centralization	Characteristic path length	Network diameter	miRNA with highest degree in the network
Luminal-A gain	3.5	38.65	0.073	0.342	3.5	0.090	0.121	2.982	6	miR-26b
Luminal-A loss	2.9	63.24	0.185	0.125	2.9	0.054	0.136	3.724	9	miR-145
Basal-like gain	2.0	48.96	0.080	0.057	2.0	0.037	0.077	4.229	11	let-7e
Basal-like loss	2.4	35.98	0.088	0.039	2.4	0.046	0.092	3.943	11	miR-21

**Table 3:** The KEGG and CePa pathway enrichment analysis results for the four gene groups.

Gene group	KEGG pathway enrichment analysis	CePa pathway enrichment analysis
Luminal-A gain	hsa05212: pancreatic cancer (*P* = 0.0093)	hsa05212: pancreatic cancer

Luminal-A loss	hsa03440: homologous recombination (*P* = 0.0013)	—
	hsa05200: pathways in cancer (*P* = 0.0096)	—

Basal-like gain	hsa00250: alanine, aspartate, and glutamate metabolism (*P* = 0.0052)	hsa00250: alanine, aspartate, and glutamate metabolism
	hsa04060: cytokine-cytokine receptor interaction (*P* = 2.85*E* − 04)	hsa04060: cytokine-cytokine receptor interaction
	hsa04810: regulation of actin cytoskeleton (*P* = 0.0036)	—
	hsa04630: Jak-STAT signaling pathway (*P* = 4.73*E* − 05)	—
	hsa05130: pathogenic *Escherichia coli* infection (*P* = 0.0095)	—

Basal-like loss	hsa04142: lysosome (*P* = 0.0033)	—
	hsa04512: ECM-receptor interaction (*P* = 0.0061)	—
