# Supplementary material for: Identification of Novel Breast Cancer Subtype-Specific Biomarkers by Integrating Genomics Analysis of DNA Copy Number Aberrations and miRNA-mRNA Dual Expression Profiling
Source: Biomed Res Int. 2015 Apr 15;2015:746970. doi: 10.1155/2015/746970 (PMC4413257; doi:10.1155/2015/746970)

Supporting Information

Table S1. The oligonucleotide sequences

Criteria	Gene	Sequence (5'-3')
siRNA	siBRCA1 target	GGAACCUGUCUCCACAAAG
qRT-PCR	BRCA1	Forward: GGAACTAACCAAACGGAGCA
		Reverse: TAGGTTTCTGCTGTGCCTGA
	β-actin	Forward: TCACCCACACTGTGCCCATCTACGA
		Reverse: CAGCGGAACCGCTCATTGCCAATGG
	pri-miR-143	Forward: TCCCCTCTAACACCCCTTCTC
		Reverse: CACACTCCTCCTGCCCAAGA
	pre-miR-143	Forward: TGAGGTGCAGTGCTGCATC
		Reverse: GCTACAGTGCTTCATCTCAGACTC
	pri-miR-145	Forward: TGGATTTGCCTCCTTCCCA
		Reverse: TTGAACCCTCATCCTGTGAGCC

	pre-miR-145	Forward: CTTGTCCTCACGGTCCAGTT
		Reverse: ATTTCCAGGAATCCCCATCT
	hRNU6-1b	Forward: CTCGCTTCGGCAGCACA
		Reverse: AACGCTTCACGAATTTGCGT
pmirGLO	miR-143	Forward: GCGAATTCGCGGAGCAGCGCAGCGCCCT
		Reverse: GCGTCGACTGGCTGCAGAACAACTTCTCT
	miR-145	Forward: GCGAATTCGGCGGCCTTGGCGCTGAAGGCCAC
		Reverse: GCGTCGACTGGGGTGGGAAGGAGGCAAATCCA

Figure S1 The Bayesian network construction process

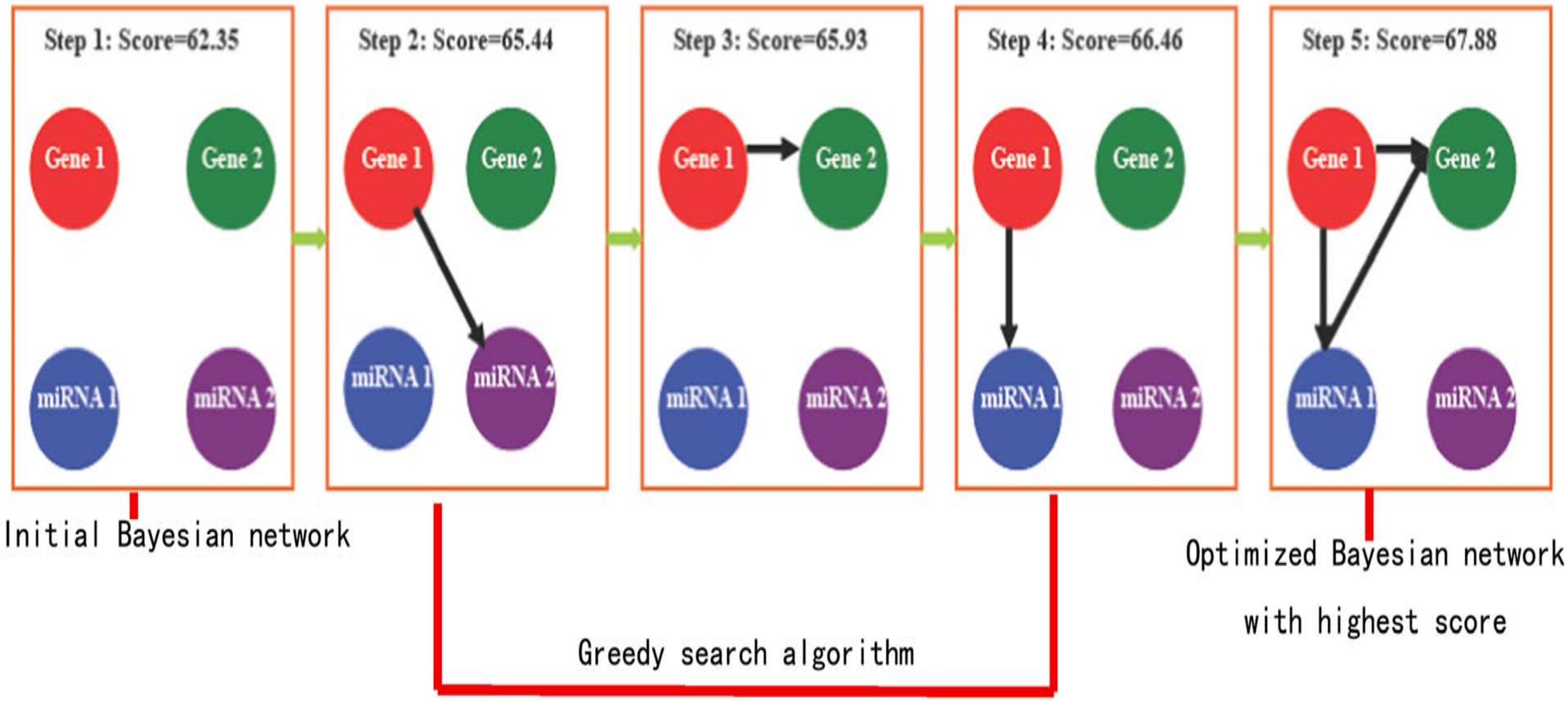

Figure S2 The highest counts of CNA regions for a luminal-A subtype and a basal-like subtype

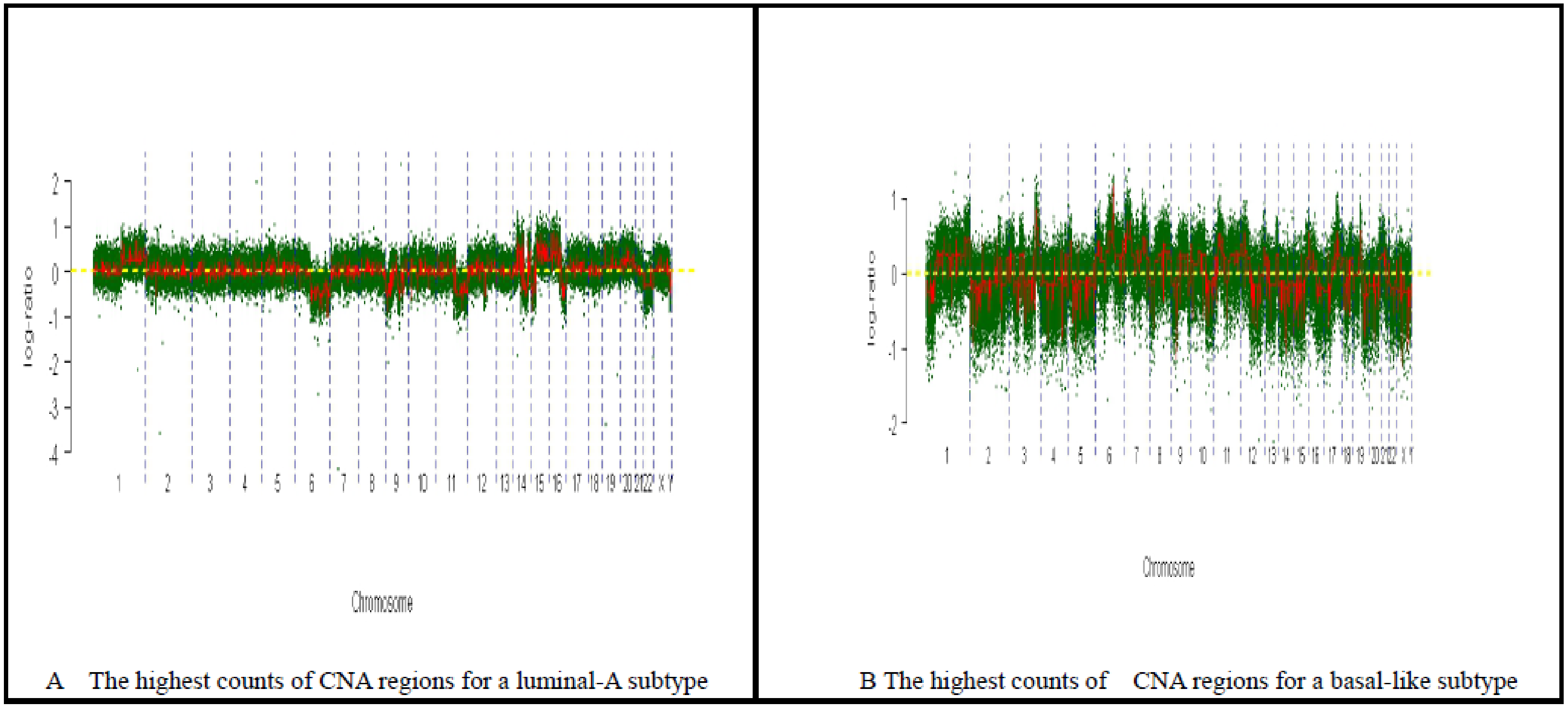

A The highest counts of CNA regions for a luminal-A subtype

B The highest counts of CNA regions for a basal-like subtype

(A) The highest counts of CNA regions for a luminal-A subtype (B) The highest counts of CNA regions for a basal-like subtype.

Figure S3 The average gene expression of four gene groups

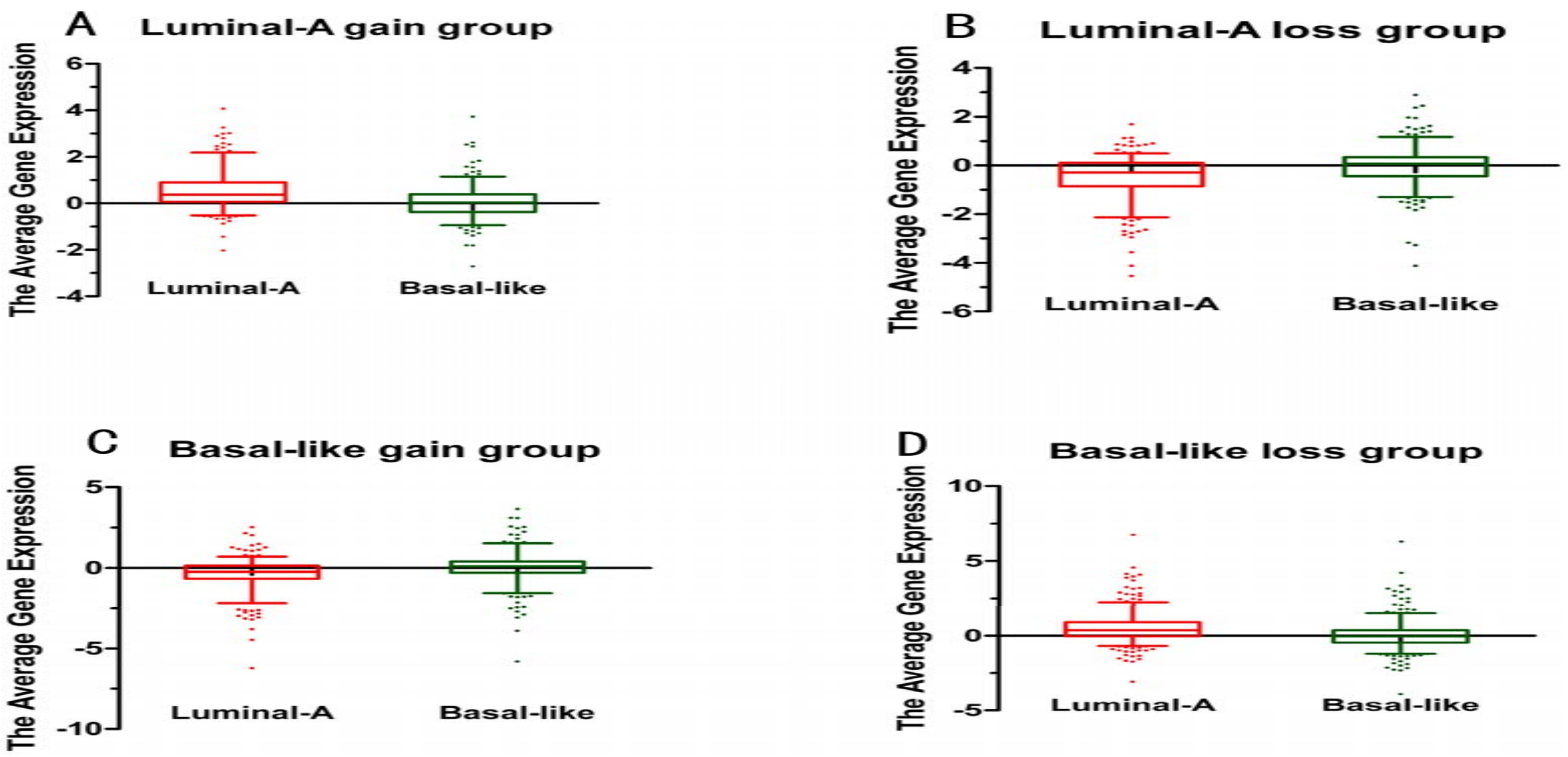

Note: The Average Gene Expression indicates the average expression value of genes involved in the corresponding sample group.

Figure S4 Kaplan-Meier (KM) survival analysis for genes involved in the identified network motifs

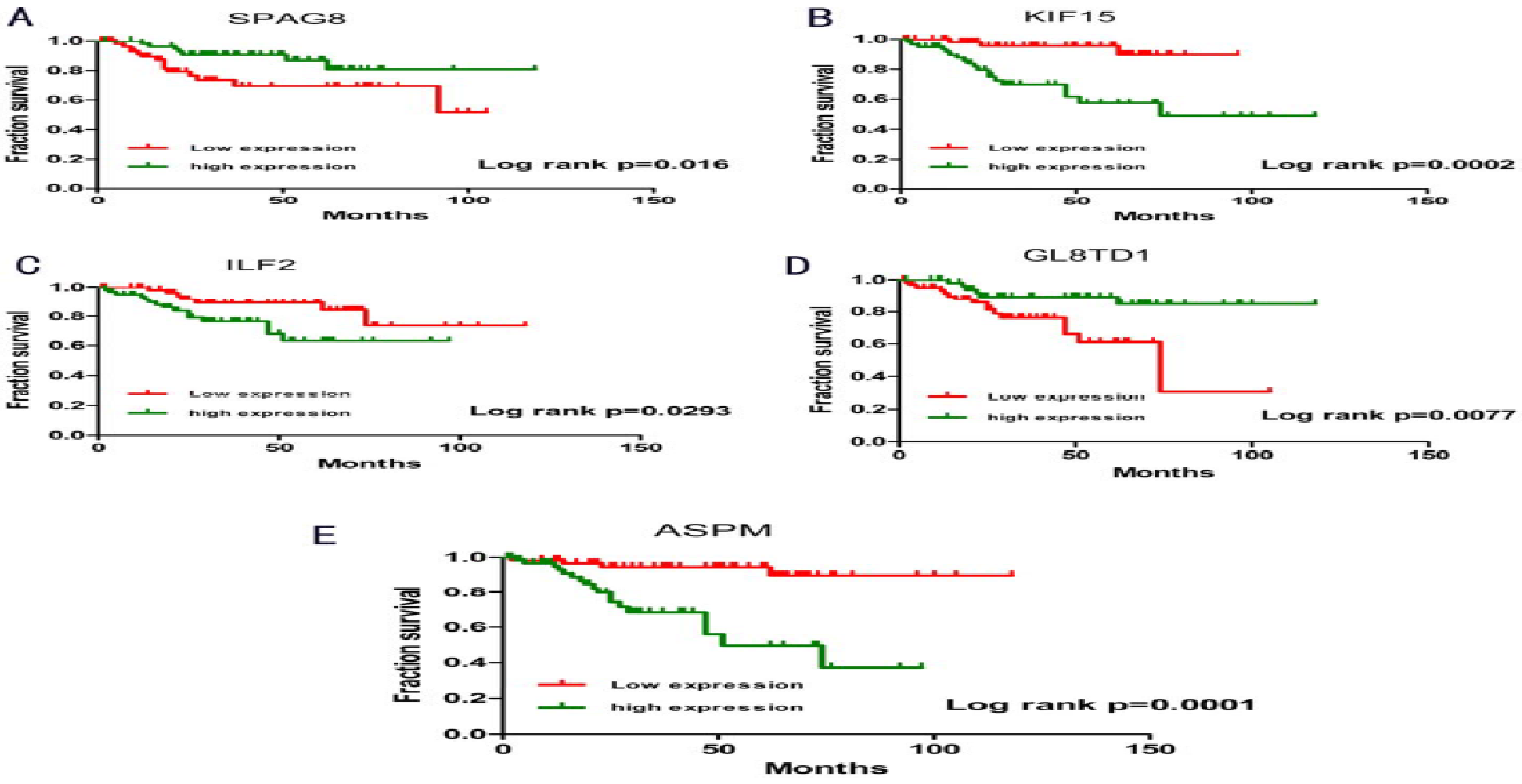

Supplement: Supplementary file 1 — Figure S1 provided the dynamic Bayesian network construction process. Figure S2 provided the highest counts of CNA regions for a luminal-A subtype sample and a basal-like subtype sample. Figure S3 is the box plots displaying the average gene expression of four gene groups (luminal-A gain, luminal-A loss, basal-like gain and basal-like loss). Figure S4 provided the Kaplan-Meier survival analysis results for genes involved in the identified four network motifs. Table S1 provided the siRNA target and the primer sequences used for cloning. [file 746970.f1.pdf]
